# Dear Editor-in-Chief Iranian J Parasitol

**Published:** 2012

**Authors:** SH Gholami

**Affiliations:** Department of Parasitology & Mycology, Faculty of Medicine, Mazandaran University of Medical Science, Iran

I received Iranian Journal of Parasitology (Volume 6 (4), Oct-Dec 2011). I have seen an article in pages 51-58 titled “Molecular and Seroepidemiological Survey of Visceral Leishmaniasis among Humans and Domestic Dogs in Mazandaran Province, North of Iran ”.

I think this title needs to be revised, because this study was done in two limited districts of the central zone of Mazandaran (Semeskandeh near Sari City (3-5 kilometer) and Kiakola district near Qahmashare City. Therefore, this study can not be considered as a study for the entire Mazandaran Province (Please see material and methods). The authors must have written the title according to the study area, because it is important for the future studies. You know, Mazandaran has a different climate and ecological conditions which effects on the species and the distribution or transmission of parasitic agents in different areas. In addition, I think in this article the previous studies in this province have not been considered properly.

**Sincerely**

**SH Gholami (PhD)**

**Assistant Professor**

**Department of Parasitology & Mycology**,

**Faculty of Medicine, Mazandaran University of Medical Science, Iran**

**Email:** sgholami@mazums.ac.ir

